# Hepatitis C Virus Glycan-Dependent Interactions and the Potential for Novel Preventative Strategies

**DOI:** 10.3390/pathogens10060685

**Published:** 2021-06-01

**Authors:** Emmanuelle V. LeBlanc, Youjin Kim, Chantelle J. Capicciotti, Che C. Colpitts

**Affiliations:** 1 Department of Biomedical and Molecular Sciences, Queen’s University, Kingston, ON K7L 3N6, Canada; e.leblanc@queensu.ca (E.V.L.); 15yk15@queensu.ca (Y.K.); c.capicciotti@queensu.ca (C.J.C.); 2 Department of Chemistry, Queen’s University, Kingston, ON K7L 3N6, Canada; 3 Department of Surgery, Queen’s University, Kingston, ON K7L 3N6, Canada

**Keywords:** hepatitis C virus, viral entry, glycans, vaccines, prophylaxis

## Abstract

Chronic hepatitis C virus (HCV) infections continue to be a major contributor to liver disease worldwide. HCV treatment has become highly effective, yet there are still no vaccines or prophylactic strategies available to prevent infection and allow effective management of the global HCV burden. Glycan-dependent interactions are crucial to many aspects of the highly complex HCV entry process, and also modulate immune evasion. This review provides an overview of the roles of viral and cellular glycans in HCV infection and highlights glycan-focused advances in the development of entry inhibitors and vaccines to effectively prevent HCV infection.

## 1. Introduction

Hepatitis C virus (HCV) is an enveloped positive-sense single-stranded RNA virus in the *Flaviviridae* family [1]. HCV is highly heterogeneous with six major genotypes and multiple subtypes identified, with distinct geographical patterns [2]. Overall, more than 70 million individuals worldwide are chronically infected with HCV [2], leading to chronic liver disease that can progress from hepatitis to cirrhosis and hepatocellular carcinoma (HCC). The HCV genome encodes one polyprotein precursor of ~3000 amino acids, processed into three structural proteins (core protein and glycoproteins E1 and E2) and seven nonstructural (NS) proteins (p7, NS2, NS3, NS4A, NS4B, NS5A and NS5B) [1]. E1 and E2 form a heterodimer complex on the viral particle, with E2 harboring the receptor binding domain (RBD) that interacts with entry receptors [3,4]. Furthermore, E2 is the major target of neutralizing antibodies [5].

The current standard of care is genotype-dependent, but usually consists of a combination therapy of direct acting antivirals (DAAs), providing safer and more efficacious treatment than previous regimens of pegylated interferon and ribavirin [6]. The advent of DAA therapy for HCV has resulted in remarkable cure rates of >90%, yet some challenges remain, such as the high cost of treatment, the potential of hepatitis B virus (HBV) reactivation in HBV/HCV co-infected individuals [7,8,9], and other difficult-to-treat patients, particularly in late stage liver disease with a non-negligeable rate of HCC recurrence [10]. Moreover, there are still no vaccines or prophylactic strategies to prevent HCV infection, and currently liver transplantation is inevitably followed by infection of the liver graft.

Early studies of HCV were challenged by the lack of a robust HCV cell culture system, although certain aspects of HCV biology, entry, and replication could be investigated using recombinant viral protein expression, lentiviral particles pseudotyped with HCV glycoproteins E1 and E2 (HCVpp) [11], and replicon systems [12]. The establishment of a fully permissive HCV cell culture (HCVcc) system [13], over a decade after molecular cloning of the HCV genome, enabled new investigations in HCV research and provided more robust insight into the virus-host interactions. HCV is unique in its association with host lipoproteins and its close relationship with lipoprotein metabolism. The formation of lipoviroparticles explains the observed heterogeneity and atypically low buoyant density of viral particles from patient serum or cell culture [14]. Apolipoproteins, such as apolipoprotein E (ApoE), play key roles in HCV entry, assembly, and production.

HCV entry is regulated by an array of host receptors and co-receptors [15]. Certain host factors are involved in HCV attachment, particularly heparan sulfate proteoglycans (HSPGs) and potentially the low-density lipoprotein receptor (LDL-R). Subsequent HCV entry steps are mediated by other host factors, including the tetraspanin CD81, the scavenger receptor SRB1, tight junction proteins claudin-1 (CLDN1) and occludin (OCLN), epithelial growth factor receptor (EGFR) and the Niemann–Pick type C1-like 1 (NPC1L1) cholesterol uptake receptor [16]. Glycan-protein interactions are also essential for many aspects of HCV entry and infection. Not only do cellular glycans, like HSPGs, act as HCV co-receptors, but virion-associated glycans also play important roles in engaging with host factors, as well as modulating host immune responses. The disruption of glycan-dependent interactions is thus an attractive antiviral approach to prevent infection. This review describes the roles of viral and cellular glycans in HCV infection and explores novel strategies that leverage our current understanding of glycan-dependent interactions to overcome the unmet challenge of preventing HCV infection.

## 2. Viral Glycans

Viral envelope proteins from various human pathogens are extensively glycosylated, and viruses exploit host cell machinery to glycosylate their proteins during replication [17]. Viral glycans, such as those found on HCV E1 and E2, have diverse and crucial roles in virus replication and virulence [17,18].

### 2.1. Glycan Profiling

**Figure 1:**
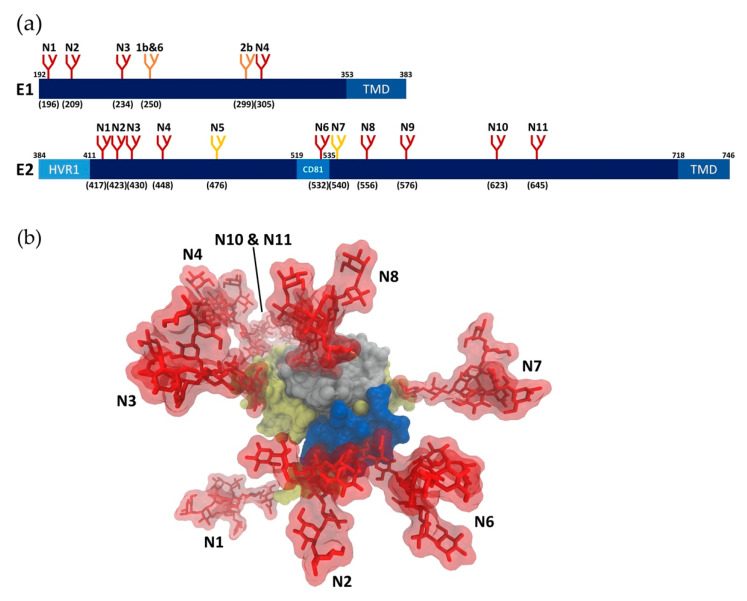
N-glycosylation of HCV glycoproteins. (**a**) Schematic representation of N-glycosylation sites on HCV E1 and E2, indicated with the letter ‘N’ followed by a number indicating the relative position of the glycosylation site in the sequence. The numbers in brackets represent the position of the glycosylation sites according to the HCV genotype 1a consensus sequence (GenBank: AF009606.1). Sites conserved across all genotypes are shown in red. Genotype-specific E1 glycans are shown in orange. E2 N-glycans are conserved across most genotypes, with the exception of genotype 1b (E2N5) and genotypes 3 and 6 (E2N7), which are shown in yellow. HVR1: Hyper variable region 1, CD81: CD81 binding loop, TMD: transmembrane domain. (**b**) Glycan shielding of E2. The model was generated in VMD by overlapping the E2 core structure (PDB:4mwf) and its N-terminal antigenic region 412–423 (PDB:4dgy). The hyper-variable region 1 is not included. The surface of E2 is shown in gray, the CD81 binding domain is highlighted in blue, and antigenic domains are highlighted in yellow. High-mannose N-glycans (Man9GlcNAc2) were modeled at 9 N-glycosylation sites (N1, N2, N3, N4, N6, N7, N8, N10, and N11) with the Glycoprotein Builder tool of GLYCAM with an energy minimization. The N-glycans are displayed in red sticks with their transparent molecular surface. N5 and N9 were not able to be installed due to the lack of sequence within the crystal structure.

HCV E1 and E2 proteins are heavily N-glycosylated in their N-terminal ectodomains, with glycans accounting for about one-third of the heterodimer mass. N-glycosylation sites on E1 and E2 are highly conserved across most genotypes (Figure 1a), indicating that glycans have critical roles in HCV infection [19,20]. It has been demonstrated experimentally that all of the conserved N-glycosylation sites are highly occupied [21,22,23]. In addition to these shared glycosylation sites, further glycosylation sites have been reported in patients and cell culture [24,25], suggesting that glycans enable HCV to adapt under selection pressure. Until recently, accurate analysis of HCV glycoproteins was challenging due to the lack of an effective cell culture system for HCV [26]. Previously, recombinantly expressed envelope glycoproteins or HCVpp were used to investigate HCV glycans and their roles in infectivity [20,27,28,29,30,31]. While these studies significantly contributed to the understanding of HCV glycosylation, the establishment of the HCVcc system in 2005 enabled analysis of glycans on the closest approximation of authentic HCV glycoproteins [26]. Notably, significant differences in E2 glycoforms have been observed in HCVpp and HCVcc systems [23,27,29]. HCVcc virion-associated E2 contains both high mannose and complex type N-glycans, whereas mainly complex type N-glycans were observed on HCVpp-associated E2 [23], reflecting differences in the assembly process of HCVcc and HCVpp. In the HCVcc system, E1E2 glycoproteins are assembled with other viral components in proximity to the ER-derived replication organelle, which could restrict access of glycan processing enzymes located in the Golgi compartment, resulting in high mannose type N-glycans [32]. Conversely, assembly of HCVpp occurs in multivesicular bodies, a post-Golgi compartment, where E1 and E2 (in the absence of other HCV components) are more accessible to glycan processing enzymes [33]. Moreover, HCVcc virions are produced in Huh7-derived human hepatoma cell lines, where the virion assembly and secretion process overlaps with the very-low density lipoprotein (VLDL) secretion pathway [34]. In contrast, HCVpp are produced in the non-hepatic 293T cell line derived from human embryonic kidney 293 (HEK293) cells, which lack the lipoprotein secretion pathway. The association of HCVcc, but not HCVpp, with lipoproteins during assembly, maturation and egress may contribute to differences in HCVcc and HCVpp glycoforms.

Glycan profiling is commonly performed by lectin microarray and mass spectrometry (MS) analyses. Lectin microarray exploits the binding specificity of a panel of lectins (glycan-binding proteins) to detect the presence of specific glycan epitopes, thus enabling the precise identification of partial glycan structures. A lectin microarray screen with HCVcc glycoproteins identified high mannose type N-glycans as the predominant glycoforms, followed by hybrid/complex type N-glycans [35]. The identified complex N-glycan glycoforms were highly heterogeneous, including abundant sialylation, fucosylation, and bisecting N-acetylglucosamine (GlcNAc). In addition, positive binding signals were detected with T antigen-binding lectins, consistent with the presence of O-glycans. A complementary MS analysis was performed to determine the oligosaccharide composition of N-glycans released from HCVcc glycoproteins after PNGase F enzymatic treatment. The 16 distinct N-glycans identified on HCVcc virions by MS were mostly consistent with the results from the lectin microarray. The microarray screen identified 10 lectins that bound to glycoproteins bearing terminal galactose (Gal) or N-acetylgalactosamine (GalNAc) residues, whereas MS identified one structure that was a mono-galactosylated N-glycan [35]. This suggests that the lectin binding to Gal/GalNAc-containing glycans may recognize O-glycans bearing these epitopes, although this has not been fully investigated. The presence of O-glycans was observed in MS analysis of recombinant E2 [31], but was not reported in MS analysis of E2 derived from HCVcc [36]. It is possible that O-glycosylation is genotype-specific, but there is currently little evidence to support O-glycosylation of E1E2 glycoproteins. Identification of HCV E1E2 glycans by glycan profiling expands our understanding of the mechanisms of viral entry and roles of glycans in HCV infection, which may provide novel strategies for therapeutics or vaccines.

### 2.2. Glycan Functions

Glycosylation is important for multiple aspects of HCV infection, including folding and assembly of envelope glycoproteins, infectivity of viral particles, and evasion of antibodies. Site-directed mutagenesis has been utilized to selectively remove N-glycosylation sites in E1E2 to study their individual roles. Studies using HCVcc showed that E1N1, E2N8, and E2N10 glycans have roles in HCV assembly. E2N3 and E2N7 glycans have a vital role in viral entry, while E2N1, E2N2, E2N4, E2N6, and E2N11 glycans contribute to immune evasion [23]. However, studying the roles of glycans on HCV envelope proteins by removal of specific glycosylation sites is challenged by the inherent importance of several glycans in proper E1/E2 folding and infectivity of HCV, and genotype differences in the tolerability of mutation sites [22,23]. Moreover, discrepancies between HCVcc and HCVpp systems have been reported.

#### 2.2.1. Glycans in Viral Entry & Infectivity

Glycans on E1 and E2 modulate viral entry and infectivity by influencing the binding of envelope glycoproteins to cellular receptors. For example, removal of the E2N1 and E2N6 glycosylation sites increased HCVcc infectivity, perhaps as a result of reduced steric hindrance at the CD81 binding site, which plays a critical role in HCV attachment and entry [23,37,38]. Interestingly, loss of the E2N6 site has been observed among naturally occurring HCVcc variants adapted to cell culture [39,40]. In contrast, removal of the E2N7 glycosylation site resulted in a strong decrease in HCVcc infectivity without affecting viral particle secretion, indicating that the glycan present at this position modulates virus entry [23]. The role of the E2N7 glycan in virus entry is likely genotype-specific, since this glycan site is absent in genotypes 3 and 6. Another study reported that the removal of glycans at positions E2N2, E2N8, E2N10, and E2N11 dramatically decreased infectivity [41]. Notably, in some reports, removal of specific glycans had a different effect on viral entry and neutralization sensitivity in HCVcc and HCVpp systems. For instance, E2N2 or E2N4 mutations affected HCVcc infectivity only to a minor degree, but abolished HCVpp infectivity [23]. This might be due to the differences in the assembly process mentioned above, and/or the association of HCVcc with lipoproteins [42,43]. A direct comparison between HCVcc and HCVpp systems revealed no correlation in specific infectivity, but there was a strong positive correlation in antibody neutralization [44].

#### 2.2.2. Glycans in Immune Evasion

Glycans associated with viral envelope proteins are synthesized by the host cell and recognized as self. Therefore, many viruses exploit glycosylation to evade host immune responses [18,20,23,37]. Glycan shielding, which serves to mask neutralizing epitopes from the host immune system [17], is a feature shared among many viruses, including human immunodeficiency virus (HIV), influenza, coronaviruses and HCV. Indeed, glycans play critical roles in reducing the immunogenicity of the envelope proteins and modulating sensitivity to neutralizing antibodies [45]. Compared with other viruses, glycan shielding in HCV evolves at a much slower rate, likely due to additional roles of glycans in the HCV life cycle [19,46,47]. The E2 glycoprotein, especially the CD81 binding site, is the primary target of humoral immune responses against HCV [5], along with some regions in the E1E2 heterodimeric complex [48,49,50], which suggests that these epitopes would be protected by glycans. Consistently, a crystal structure of HCV E2 showed that 7 of the 11 N-linked glycans generate an extensive glycan shield on the exposed face on the E2 surface, potentially masking E2 neutralizing epitopes, including the CD81 binding site (Figure 1b) [38]. Consistently, the removal of specific N-glycans (E2N1, E2N2, E2N4, E2N6 and E2N10) near the CD81 binding site increased the sensitivity of HCVcc to neutralization [23,51]. Despite the differences in glycoforms between HCVcc and HCVpp, the effects of removing glycans on neutralization sensitivity were highly consistent between the two systems, implying the type/size of N-glycans does not drastically affect recognition of neutralizing epitopes [22,37,44]. Furthermore, despite the conserved nature of HCV glycosylation sites, several cases of glycan shift mutations and additional glycosylation sites were reported, which alter or abolish neutralization by specific antibodies [24,45,52]. On the other hand, some studies reported that the presence of certain glycans on E2 are crucial for the binding of some neutralizing antibodies as they may be directly involved in the interactions [53,54]. For instance, the enzymatic cleavage of E2 glycans reduced binding affinity of a particular antibody, suggesting glycans maybe essential for certain E2-neutralizing antibody interactions [54]. Unlike E2 glycosylation, it was reported that E1 glycosylation does not influence the neutralization sensitivity of HCVpp to antibodies from HCV positive patient sera [20], or the infectivity and neutralization sensitivity of HCVcc [23]. However, this may be attributed to the fact that antibodies induced by immune responses against HCV are predominantly anti-E2, and the availability of anti-E1 neutralizing antibodies is very limited [55,56]. Further studies with anti-E1 neutralizing antibodies are required to identify potential roles of E1 glycans in HCV immune evasion.

#### 2.2.3. Role of Glycans in the Envelope Breathing Model

The hypervariable region 1 (HVR1) of E2 has been implicated in the protection of cross-genotype conserved epitopes from neutralizing antibodies, and HVR1 deletion mutants are more susceptible to neutralization, validating the role of HVR1 in viral immune evasion [57,58,59,60]. Neutralization sensitivity was increased by the deletion of HVR1 and further enhanced by removing specific glycans [61]. Previously, it was widely accepted that the steric hindrance from HVR1 and glycans drive epitope shielding and antibody evasion [47]. However, the concept of virus “envelope breathing”, where there is a modulation of antibody binding epitopes that can occur via conformation changes, has been reported for some flaviviruses [62,63]. This has also been suggested for HCV, based on time- and temperature-dependent neutralization properties of HCV [64]. Envelope breathing in HCV was thought to be limited due to the presence of multiple disulfide bonds in envelope glycoproteins. These disulfide bonds were suggested to restrict the breathing motion [65] and the resistance of HCV virions to acid inactivation, suggesting a lack of irreversible conformation changes reported in many other flaviviruses [66]. However, a recent study has shown that HVR1 and glycans indirectly modulate the equilibrium of glycoproteins between open and closed conformation. This results in stabilization of the neutralization-resistant closed conformation, rather than protecting neutralizing epitopes by direct steric hindrance [41]. These findings support an envelope breathing model for HCV, where the removal of glycans and HVR1 shifts the equilibrium by destabilizing the closed conformation of the envelope proteins, rendering the virus more susceptible to antibody neutralization. The envelope breathing model is supported by the observations that steric hindrance alone cannot explain the following: (1) the effect of removing glycans on neutralization sensitivity is dependent on HVR1; (2) the removal of glycans can be both neutralization sensitizing and protective; (3) the effect and magnitude of glycan removal on neutralization sensitivity are not epitope-specific; and (4) some glycans previously speculated to mask E2 epitopes are not in close proximity to the neutralization epitopes in recent crystal structures of E2 [20,23,38,67]. At this point, however, the envelope breathing model of HCV can only be inferred from antibody reactivity studies. It is challenging to elucidate the precise structural rearrangements involved due to limitations of structural studies and understanding the dynamic properties of glycoproteins from enveloped viruses such as HCV [68,69,70]. Further studies are required to fully understand the roles of glycans in antibody evasion during HCV infection and to understand the dynamic conformations of HCV envelope proteins.

### 2.3. Viral Glycan Interactions with Cellular Lectin Receptors

Many cell surface lectins interact with E1 and E2 or have been shown to play roles in HCV entry, including dendritic cell-specific intercellular adhesion molecule-3-grabbing nonintegrin (DC-SIGN) and liver/lymph node-SIGN (L-SIGN; also known as CLEC4M, DC-SIGNR, CD 209L) [71,72,73], asialoglycoprotein receptor (ASGPR) [74], L-ficolin [75,76], liver sinusoidal endothelial C-type lectin (LSECtin) [77] and mannose binding lectin (MBL) [78]. 

**Figure 2:**
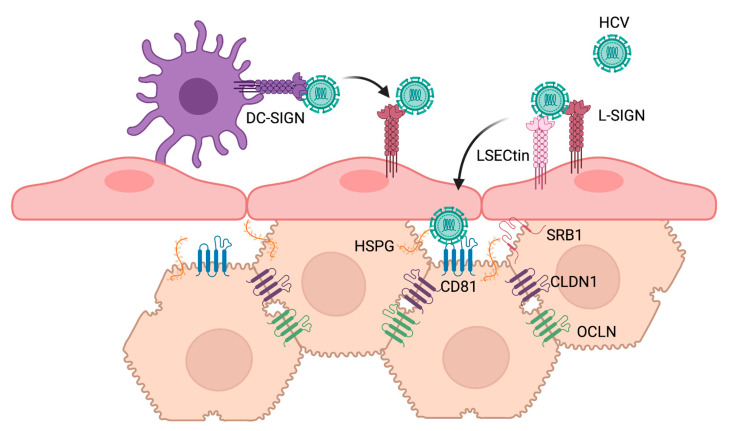
Putative roles of lectin receptors in HCV capture. The current evidence is consistent with a model where HCV virions are captured by dendritic cell (purple) lectins, such as DC-SIGN, carrying the virus through the blood to the liver. In the liver, HCV particles are transferred to liver sinusoidal endothelial cells (LSECs, pink) via L-SIGN and potentially LSECtin, and are finally transferred to hepatocytes (beige), which express the required attachment and post-attachment receptors for HCV infection. Created with Biorender.com.

Soluble E2 as well as HCVpp were demonstrated to bind to cells induced to express DC-SIGN and L-SIGN, but not other C-type lectins such as CD23, CLEC-1, or CLEC-2 [71]. E2 binding to cell surface SIGN proteins was inhibited by mannan, which competes for binding to the lectin domain of the receptors, as well as by EGTA, which chelates calcium ions required for the carbohydrate-binding properties of these lectins [71,72,73]. Deglycosylation of E2 by PNGase F treatment reduced binding to both lectins. Interestingly, E2 was unable to interact with immobilized distant monomeric carbohydrate recognition domains (CRD) of DC-SIGN and L-SIGN lectins, but high affinity binding was observed with closely seeded CRDs, particularly when oligomerized, similar to how they would be found multimerized in the extracellular domain of DC-SIGN [73]. Together, these early studies provided evidence for the role of multivalent interactions by clusters of high-mannose N-glycans on E2 as binding motifs for interaction with DC-SIGN and L-SIGN receptors [73]. Importantly, HCV derived from the serum of infected patients was shown to bind cells expressing L-SIGN and DC-SIGN, and this binding was also competitively inhibited by mannan [72]. HCVpp and serum-derived HCV may also bind to cells expressing LSECtin [77], although its relative contribution to HCV binding remains controversial [79,80]. It is thought that L-SIGN, and potentially LSECtin, expressed on liver sinusoidal endothelial cells (LSECs) and DC-SIGN expressed on dendritic cells act as capture receptors. This hypothesis is supported by co-culture experiments in which infective virus is transmitted from L-SIGN and DC-SIGN-expressing cells that are refractory to HCV infection, to permissive Huh7 cells [81,82,83]. The binding affinity of E1E2 for several lectin and non-lectin receptors was directly measured by surface plasmon resonance to help elucidate the relative preference of the HCV glycoprotein complex for its many interacting receptors. This study revealed that E1E2 proteins bind lectin receptors DC-SIGN and L-SIGN with very high affinity relative to non-lectin receptors [80]. When co-transfected in HEK293T cells, LSECtin interacted with L-SIGN as well as, albeit less strongly, the canonical HCV receptor CD81 [77]. It has been proposed that HCV particles are captured by immune cell lectins, such as DC-SIGN, carrying the virus through the blood to the liver, where the virus then interacts with LSECs via L-SIGN and LSECtin. HCV particles are then ultimately transferred to hepatocytes, which express the required co-receptors for HCV infection (Figure 2) [80]. 

Opposing roles are proposed for other lectins. High concentrations of L-ficolin have been detected in the serum of HCV patients [75,76]. L-ficolin, as well as MBL, were found to bind HCV glycoproteins and to activate the lectin complement pathway [75,78]. Additionally, both MBL and L-ficolin neutralize HCV entry as demonstrated by specific, dose-dependent inhibition of HCVpp [76] and HCVcc entry [78]. L-ficolin and MBL appear to interact with HCV via N-glycans on its glycoproteins and antagonize infection through multiple mechanisms. Knowledge of the roles of various lectin receptors in HCV infection has helped to dissect the complex mechanism underlying hepatocyte infection by HCV and has opened perspectives for novel antiviral preventive strategies.

## 3. Cellular Glycans

Like other *Flaviviridae*, HCV attachment is mediated by interactions with cell surface HSPGs. It was initially thought that this interaction relied on binding of the HCV envelope protein E2 to cellular HSPGs [84]. The E2 hypervariable region-1 (HVR1) contains positively charged residues and was demonstrated to be required for the high affinity binding of recombinant E2 to heparin, a linear highly sulfated structural homolog of HSPGs [84]. Further studies with recombinant E2 determined that binding required heparin with N-sulfation and a minimum length of 10 monosaccharide subunits [85]. At the same time, another study using HCVpp and HCVcc found O-sulfation to be critical [86]. 

However, as the role of ApoE in HCV infection emerged, it became evident that virion-associated ApoE binds HSPGs and mediates HCV attachment. Jiang et al. observed that treatment with an ApoE-monoclonal antibody potently blocked HCV attachment, but did not inhibit infection when HCV was already bound to cells, while an HCV-E2 antibody had the opposite effect [87]. ApoE has a C-terminal lipid binding domain and an N-terminal receptor binding domain (RBD), the latter having positively charged amino acids that interact with HSPGs. Site-directed mutagenesis that disrupted the heparin binding capacity of ApoE reduced HCV infection, consistent with a role for virion-associated ApoE in HCV attachment to HSPGs [87,88]. Furthermore, synthetic peptides composed of 18 and 21 amino acids that were derived from the ApoE N-terminal RBD blocked HCV attachment [88] and infection [87]. Cell binding studies, competition assays and heparin pulldown experiments demonstrated that the E2 HVR1 is not the main determinant for cell surface attachment of HCV virions to HSPGs. However, isolated HVR1 can bind HS, consolidating the biochemical and functional observations [89]. Findings with HCV virions confirm that a decasaccharide is the minimum heparan sulfate (HS) oligosaccharide length and that sulfation pattern, including N- and 6-*O*-sulfation, but not 2-*O*-sulfation, are critical for HCV infection [89]. Notably, liver HSPGs have a high level of sulfation relative to other tissues [90,91]. 

A variety of proteoglycans are produced by human cells, but the major cell surface HSPGs fall into two groups: syndecans (SDCs) and glypicans (GPCs). To determine the importance of HSPG core proteins, Shi et al. silenced expression of four human SDCs and six GPCs, and found that only SDC1 and SDC2 reduced HCVcc attachment [92]. SDC1 silencing reduced infectious HCV titers by nearly 100-fold. Furthermore, silencing SDC1 expression in human embryonic stem cell-differentiated human hepatocytes reduced attachment of an HCV genotype 1b clinical isolate, supporting the physiological relevance of SDC1 in HCV attachment [92]. These RNAi-based studies were later supported by CRIPSR knockouts of SDC1-4, where only the absence of SDC1 and SDC2 significantly reduced HCVcc attachment and infection [93]. Independently, others reported that silencing of SDC4 reduced HCVcc infectivity [88]. However, a modest reduction in HCVpp infectivity was also observed in SDC4 knock-down cells, and since HCVpp are produced independently of apolipoprotein association in 293T cells, it was suggested that SDC4 may have an ApoE-independent role, possibly through interaction with E2. Interestingly, a third group performed siRNA-mediated silencing of SDC1 and SDC4 and validated the finding that silencing of SDC1 reduces infectivity of cell culture and clinical isolates of HCV, but they also observed a significant reduction in HCVpp infectivity [94]. The combined knockdown of SDC1 and the post-attachment receptor CD81 significantly reduced infection compared to either single knockdown, suggesting cooperative activity [94]. Furthermore, SDC1 was shown to interact with CD81 [94]. Finally, while cell-free transmission is the major route of HCV infection, cell-to-cell spread also occurs between neighboring cells and may contribute to the evasion of innate host immunity and persistent infection [95]. SDC1 and SDC2 were observed to have modest roles in the promotion of HCV cell-to-cell transmission, in comparison to the demonstrated importance of tight junction post-attachment receptors, CLDN1 and OCLN [93]. Collectively, these findings have paved the way for the development of antiviral strategies targeting early stages of HCV attachment. 

## 4. Therapeutics and Vaccine Development

Over the last two decades, many strategies have been explored to disrupt the glycan-based interactions required by HCV for attachment and entry. While approval of the first DAAs in 2013 revolutionized the treatment of individuals suffering from HCV infection, the development of HCV entry inhibitors remains a priority to prevent reinfection of transplanted livers in patients with advanced liver disease, as well as to develop prophylactic strategies to protect populations at highest risk of HCV infection in the absence of an HCV vaccine.

### 4.1. Targeting the HCV-HSPG Interaction

Two main approaches have been utilized to block the interaction between HCV and cell surface HSPGs: the development of glycan mimetics and ApoE mimetics. As soon as the binding requirements regarding size and sulfation of HS were elucidated, the development of HS-based antivirals to target HCV attachment was proposed [85]. Although efforts to dissect the critical sulfation patterns continued [86,89], very few advancements were made toward the development of novel HS-based compounds to block HCV infection. Future studies are warranted to evaluate various HS mimetics that have been shown to inhibit infection by other *Flaviviridae*, such as dengue virus, without the anticoagulant activity of heparin [96,97,98].

The natural product epigallocatechin gallate (EGCG) provides proof-of-concept that a small molecule compound can block HCV infectivity by inhibiting virion attachment to heparan sulfate [99]. Notably, the inhibitory activity of EGCG was demonstrated for all HCV genotypes tested and validated in primary human hepatocytes [100,101]. Structure-activity relationship studies were initiated to identify EGCG analogs with improved efficacy against HCV and improved drug-like properties, but only a limited increase in potency was achieved [102]. Nonetheless, the therapeutic potential of entry inhibitors, including EGCG and AR4A, an E1/E2 monoclonal antibody (mAb), alone and in combination were evaluated in human liver chimeric mice challenged with HCV genotype 1a [103]. While the combination was additive in vitro, no significant difference was observed between the protection offered by the mAb alone compared to in combination with EGCG in vivo. EGCG monotherapy failed to protect mice from HCV infection, possibly due to the low bioavailability of orally administered EGCG [103]. Nonetheless, EGCG has been incorporated into experimental treatment regimens. A recent pilot clinical study found that patients with genotype 4 HCV infections receiving a single tablet formulation of two DAAs in combination with 400 mg of EGCG had a significantly more rapid rate of viral load decline compared to patients receiving the standard of care therapy consisting of the same combination of DAAs but without EGCG [104]. A rapid rate in viral decline could provide an avenue for the reduction in treatment duration and therefore costs, providing greater accessibility to treatment. The new combination, Dactavira, was well tolerated and the sustained virological response for 12 weeks was no different than the standard of care [104]. These preclinical and clinical studies point to the need for further mechanistic characterization of the interaction between HCV and EGCG to design analogous small molecule entry inhibitors with improved properties to enhance treatment efficacy. 

Consistent with the role of ApoE in HCV attachment, multiple ApoE-derived synthetic peptides effectively block HCV infection [87,88,105]. The first characterization of a rationally designed human ApoE peptide (hEP) inhibited HCVcc infection with an IC_50_ of 0.67 μM, showed no cytotoxicity, and was stable in culture media for at least 24 h [105]. The lack of activity against HCVpp and other human viruses suggested on-target activity, as ApoE is uniquely required for infectivity of cell-culture- or patient-derived HCV [105]. The mechanism of hEP was further confirmed with time-of-addition and binding experiments that demonstrated activity at the attachment stage. Furthermore, pre-incubation of serum-derived HCV from five patients with a hEP analog significantly reduced virus binding to primary human hepatocytes providing physiological validation of this approach [105]. Later, as short as a duplicate of nine amino acid residues from the HSPG-binding domain was demonstrated to be effective at blocking HCV entry [88]. Notably, the systemic administration of ApoE mimetics has been reported previously to have beneficial effects, such as reduction in blood cholesterol levels and inflammation [106,107]. This distinct antiviral mechanism, with reduced likelihood for the development of resistance, warrants further in vivo investigation to evaluate the therapeutic potential of ApoE mimetics as part of combination therapy for HCV [105]. 

### 4.2. Targeting Viral Glycans

More recently, antiviral strategies have focused on targeting virion-associated glycans. E2 glycosylation sites are highly conserved across genotypes, are crucial for proper protein folding, and play roles in viral entry and immune evasion. As such, E2 post-translational glycosylation provides a potential antiviral target with a high fitness cost for mutation. Glycosyltransferase inhibitors, particularly those that disrupt N-glycan biosynthesis such as tunicamycin, disrupt glycosylation of viral glycoproteins, conferring antiviral activity. Tunicamycin-derived molecules have been developed that display broad antiviral activity with substantially reduced mammalian cytotoxicity [108,109]. Compounds with low micromolar inhibitory activity against HCV have been identified, but efforts are ongoing to improve potency and selectivity [110]. 

The development of safe and potent lectin-based compounds targeting HCV has recently shown great promise. Lectins that target high-mannose-type N-glycans (HMGs) display broad spectrum antiviral activity. While off-target activities are expected to be minimal, since normal human cells rarely have clusters of high mannose glycans, a major drawback of lectins is their propensity to induce mitogenicity. Promisingly, it has recently been demonstrated that mitogenicity can be uncoupled from antiviral activity through directed mutagenesis [111]. An engineered banana lectin (H84T BanLec) demonstrated pan-genotypic inhibition of HCV infection, as well as other viruses with HMGs, such as HIV and influenza virus [111]. Most recently, a fusion protein consisting of an HMG-targeting lectin and the Fc region of a human IgG antibody was shown to bind HCV E2 [112]. Upon further investigation, this *lectibody*, called AvFc, had low nanomolar inhibitory activity against at least five HCV genotypes tested [113]. The impressive inhibitory concentrations are partially attributed to the multivalent recognition of HMGs due to the dimerization of the lectin via the Fc fusion [113]. AvFc was formulated for in vivo efficacy testing and, remarkably, was shown to protect human liver chimeric mice against HCV infection for up to 35 days, without apparent toxicity [113]. The potent pan-genotypic activity and the efficacy of systemic administration of AvFc provides a strong rationale for evaluation of the lectibody in future trials, particularly for prophylactic use to prevent infection during liver transplant or to protect populations at high risk of HCV infection.

### 4.3. Vaccine Development

The high rate of asymptomatic infection and the risk of reinfection make the development of vaccines imperative for global control of HCV. E1E2 glycoproteins are major immunogens targeted in vaccine design, and several vaccine candidates employing various approaches showed promising results in chimpanzee models or human clinical trials [114]. However, significant challenges remain due to the high genetic diversity of HCV and other immune evasion strategies of the virus [115,116,117,118].

One of the major challenges for developing a pan-genotypic vaccine is the identification of epitopes conserved among highly diverse HCV sequences. It was shown that glycoprotein regions with high mutation rates are often targeted by humoral antibody responses [119], which reduces the possibility of cross-genotype vaccine development. Considering their highly conserved sites across HCV genotypes, targeting N-glycans has great potential for developing pan-genotypic vaccines. Glycoconjugate vaccines are very successful for bacterial infections [120], but unlike other pathogens, viruses exploit the host glycosylation machinery and decorate themselves with self-glycans, complicating the design of viral carbohydrate-based vaccines. However, the high glycan density and other viral components on the surface of most viruses are shown to limit the access of glycan processing enzymes, resulting in high-mannose type glycans that are uncommon in mammalian proteins. Although it has not been reported in the context of HCV, a broadly neutralizing antibody that binds specifically to a conserved cluster of oligomannose glycans on HIV-1 gp120 envelope glycoprotein has been reported [121,122] and it has been demonstrated to bind a wide range of HIV-1 isolates [123]. Based on this finding, there have been multiple attempts to develop glycoconjugate vaccines for HIV [124]. While the current success remains limited, efforts are still ongoing, and recent developments in glycoengineering and glyco-virology may pave the way for viral glycoconjugate vaccine design and their applications in HCV prevention.

**Figure 3:**
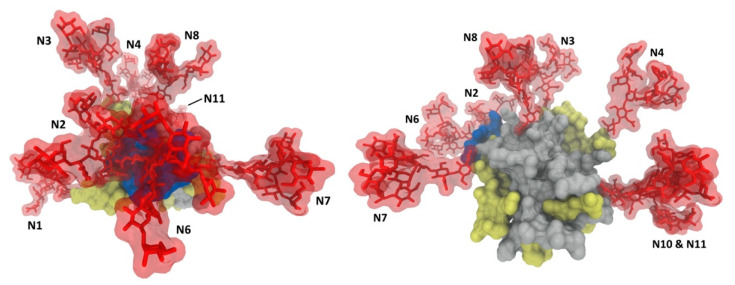
Glycan shielding of HCV E2 glycoprotein. N-glycans are extensively shielding the CD81 binding region and antigenic domains of E2 (**left**), whereas there are fewer shielding effects on the non-antigenic face of E2 (**right**).

Highly abundant glycosylation on E1E2 glycoproteins reduces immunogenicity by shielding conserved epitopes from neutralizing antibodies [20,22,23,37,47] (Figure 3). Therefore, attempts have been made to selectively engineer glycans on surface viral glycoproteins to modulate the presentation of conserved epitopes for enhanced immunogenicity. Multiple glycan modification approaches were employed, including selective deglycosylation by site-directed mutagenesis, glycan type manipulation, and hyper-glycosylation. In HCV, the deletion of specific glycan(s) on E1 or E2 glycoproteins resulted in increased binding of certain neutralizing antibodies and improved humoral and/or cellular immune responses compared to wild-type glycoproteins. In studies using DNA-based vaccination in a mouse model, the removal of specific glycosylation site(s) was shown to improve the immunogenicity of E1 and/or E2. Enhanced anti-E1 humoral and E1-specific cellular immune responses were observed by E1N4 and E1N2 mutants, respectively [125,126], and the E2N9 mutant was shown to elicit higher E2-specific cellular immune response [127]. E1E2 heterodimer N-glycosylation mutations were also explored, showing the highest cellular immune response induced by E1N2 and E2N3 combination mutations. Serum from these vaccinated mice showed effective neutralization of HCVcc genotype 2a and HCVpp genotypes 1–7, suggesting that broadly neutralizing antibodies had been elicited [128]. Another study showed that vaccination with lettuce-produced E1E2 heterodimer E2N6 mutant elicited improved immunogenic properties compared to E1E2 wild-type in a mouse model by oral vaccination [129]. Despite the great potential shown by these studies, the removal of glycans should be undertaken cautiously and must consider other vital roles of the glycans, such as in protein folding and expression, to ensure biologically relevant E1E2 immune responses. Therefore, it is critical to balance removing glycans for enhanced immunogenicity and retaining glycans for their biological functions when considering the optimal design of vaccines [47]. Moreover, removal of both specific glycans and HVR1 was investigated to evaluate the combined effects on enhancing the immunogenicity of E2 by exposing the CD81 binding site and conserved cross-neutralizing epitopes [61]. The recombinant E2 mutants showed improved binding to CD81 and antibodies targeting the CD81 binding site. However, the antibodies elicited from immunization with the mutants were cross-binding but not cross-neutralizing, and the neutralizing antibodies that were induced were strain-specific [61]. Another study also reported that the removal of HVR1 minimally affected immunogenicity in E2, evidenced by unchanged neutralizing antibody titers induced by soluble E2 vaccination in a mouse model [130]. These studies imply that epitope exposure by deleting interference may not be sufficient for inducing cross-neutralizing antibodies, likely due to the considerable overall conformational flexibility of E2, especially in the CD81 binding region [38,67,131]. It was suggested that the flexibility of this region might direct the induction of non-neutralizing antibodies [132]. Notably, a similar immune evasion mechanism has been observed for HIV [133]. Therefore, securing the rigidity of conserved epitopes should be considered in vaccine design as well as the exposure of the epitopes. This is consistent with the envelope breathing model, where the conformation changes of the glycoproteins modulate the neutralization sensitivity rather than the steric hindrance alone. 

In addition to the complete removal of specific glycosylation sites, truncation of glycan structures to increase the exposure of epitopes was investigated using various expression platforms and enzymatic modifications. Improved immunogenicity and higher induction of broadly neutralizing antibodies across a panel of HCV genotypes were reported with E2 recombinantly expressed in insect cells, which are known to have less processed paucimannose type glycans (short/truncated mannose type N-glycans) compared to human cells with mainly complex and high mannose type glycans [134]. This study suggests that the glycoform can influence the immunogenicity of E2 and should be considered for vaccine design. However, a recent study reported contradicting results, where there were no appreciable differences found in overall antigenicity, immunogenicity, and neutralization breadth in the two expression systems [135]. This suggests that increased exposure of epitopes by reducing the size of glycans does not translate to increased immunogenicity There are differences in experimental conditions in the two studies, including E2 genotypes, adjuvants and HCV systems (HCVcc vs HCVpp), which might explain these discrepancies. Thus, the extent of the influence of glycosylation patterns on antigenicity and immunogenicity remains elusive and requires further investigation. 

Another glycan-related epitope engineering strategy is hyper-glycosylation, which was employed to mask epitopes involved with non-neutralizing antibodies and direct the immune response to desirable epitopes. This strategy has been applied to HIV [136,137,138] and, recently, HCV infection [134]. It was reported that HCV infection can induce antibodies that interfere with the binding of neutralizing antibodies induced by vaccination by blocking the neutralizing epitopes [139]. Depletion of interfering antibodies can elicit cross-genotype neutralizing activity [140], which implies that hyper-glycosylation may be an effective strategy to mask epitopes for non-neutralizing/interfering antibodies in HCV. In a recent study, hyper-glycosylation was utilized to mask E2 antigenic domains associated with non-neutralizing antibodies, but no increase was observed in neutralizing antibody titers from the hyper-glycosylation mutant [130]. The mutant combined with HVR1 removal resulted in a modest improvement in neutralizing antibody titers against one of the neutralization resistant HCVpp tested, suggesting the potential for hyper-glycosylation in vaccine design [130]. 

**Figure 4:**
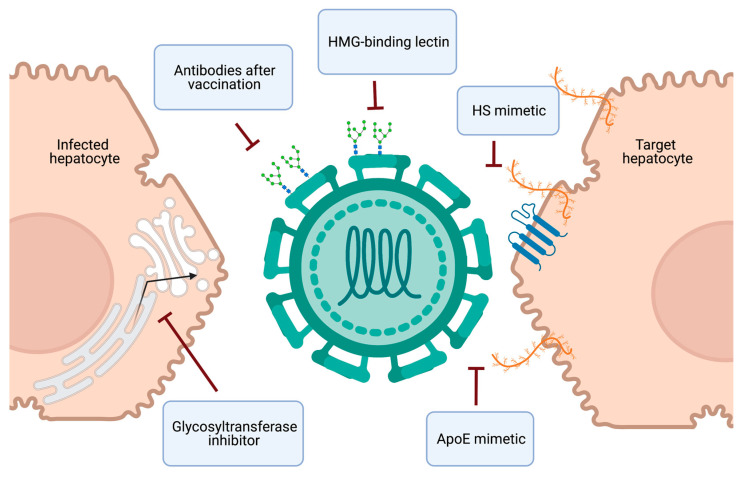
Schematic representation of HCV preventative strategies and their molecular targets. Clockwise from top: The production of antibodies in response to an immunogenic vaccine or the systemic administration of high-mannose glycan (HMG)-binding lectins could neutralize HCV. Small molecules or synthetic peptides that mimic heparan sulfate (HS) or apolipoprotein E (ApoE) respectively, could disrupt the glycan-dependent attachment of HCV preventing entry and infection of target hepatocytes. Glycosyltransferase inhibitors could inhibit the glycosylation of HCV glycoproteins, rendering progeny virions non-infectious. Created with Biorender.com.

Collectively, targeting viral or cellular glycans provides novel perspectives to prevent HCV infection through antiviral prophylaxis or vaccine development (Figure 4). 

## 5. Conclusions and Future Directions

HCV therapy has been revolutionized in the last decade due to the development of safe and effective DAAs. Moreover, the scale up of prompt HCV infection diagnosis and universal DAA treatment in high-risk settings has recently demonstrated the potential for improved infection control with a “treatment as prevention” strategy [141,142]. To enhance HCV elimination efforts, the next frontier in HCV research is the development and implementation of accessible preventative strategies (Figure 4). Naturally occurring molecules, such as heparin and EGCG, block HCV infection by inhibiting virion attachment to heparan sulfate and demonstrate the potential of this antiviral strategy. An improved mechanistic understanding of the interaction between these molecules and HCV virions is needed for the design of small molecules with improved potency and specificity. The encouraging long-lasting protective effect of a carbohydrate-binding lectin fusion protein in human liver chimeric mice [113] opens perspectives for the development of a pan-genotypic HCV entry inhibitor. As well, recombinant human lectin L-ficolin has been demonstrated to neutralize multiple HCV genotypes in vitro [76,143], further supporting the potential of a lectin-based antiviral strategy with a high barrier to resistance [144]. Therapies targeting glycan-based interactions also provide the advantage of potential broad antiviral activity, which could help resolve the issue of HBV reactivation in co-infected patients treated with current HCV therapy. The ultimate preventative strategy is the development of an effective HCV vaccine, which remains elusive. However, there is cause for optimism, as a more detailed understanding of the conflicting roles of glycans in immune evasion and immunogenicity, and advances in glycoengineering, are bolstering new strategies in HCV vaccine design.

## Data Availability

No new data were created in this review. Crystal structures presented in this article are available in the Protein Data Bank (PDB, https://www.rcsb.org/ (accessed on 26 May 2021)) entry 4mwf and 4dgy.
